# The mitigation of activity-based anorexia by obese adipose tissue transplant is abolished by neonatal AgRP neuron ablation

**DOI:** 10.1038/s41398-026-03970-2

**Published:** 2026-03-23

**Authors:** Dongmin J. Yoon, Jie Zhang, Rizaldy C. Zapata, Martina Ulivieri, Avraham M. Libster, Matthew S. McMurray, Olivia Osborn, Stephanie C. Dulawa

**Affiliations:** 1 Department of Psychiatry, University of California, San Diego, La Jolla, CA 92093 USA; 2 Division of Endocrinology and Metabolism, Department of Medicine, University of California San Diego, La Jolla, CA 92093 USA; 3 Department of Psychology, Miami University, Oxford, OH 45056 USA

**Keywords:** Neuroscience, Psychiatric disorders

## Abstract

Anorexia nervosa (AN) is an eating disorder observed primarily in girls and women, and is characterized by a low body weight, hypophagia, and hyperactivity. The activity-based anorexia (ABA) paradigm models aspects of AN, and refers to the progressive weight loss, hypophagia, and hyperactivity developed by rodents subjected to fixed-time food restriction and running wheel access. Recent metabolic studies identified white adipose tissue (WAT) as a primary location of the ‘metabolic memory’ of prior obesity, and implicated WAT-derived signals as drivers of relapse to obesity following weight loss. Thus, we examined whether an obese WAT transplant could attenuate ABA-induced weight loss in normal female mice. Recipient mice received a WAT transplant harvested from either standard chow-fed, or high-fat diet (HFD)-fed obese mice. Obese fat recipient (OFR) and control fat recipient (CFR) mice were then tested for ABA. OFR mice “survived” longer than CFR mice in the ABA paradigm, defined as maintaining 75% of their initial body weight. Next, we tested whether agouti-related peptide (AgRP) neurons, which regulate feeding-related behaviors and metabolism, mediate obese WAT transplant-induced resilience against ABA. CFR and OFR mice received either control or neonatal AgRP ablation, and were assessed for ABA. OFR intact mice maintained higher body weights than CFR intact mice during ABA, but this effect was abolished by neonatal AgRP ablation. Furthermore, ablation reduced survival in OFR, but not CFR mice. In summary, obese WAT transplants signal to AgRP neurons to protect against ABA. Obese WAT-derived factors may provide targets for treatment development in AN.

## Introduction

Anorexia nervosa (**AN**) is a complex illness mostly affecting women and girls [1], and is characterized by hypophagia, low body weight, and compulsive exercise [2–4]. AN has a lifetime prevalence of approximately 1%, and has one of the highest mortality rates of all psychiatric disorders [1]. Many pharmacological agents have been assessed in AN including antipsychotics, antidepressants, and anti-epileptics, but no treatment has demonstrated long-term efficacy for weight gain [5–7]. A recent large-scale genetic analysis reported that AN shows significant genetic correlations with metabolic traits, and should be reconceptualized as a metabo-psychiatric disorder [8, 9].

Activity-based anorexia (ABA) is a phenomenon observed ubiquitously in normal rodents and other mammals [10–12], and recapitulates several aspects of AN [13–17]. In the ABA paradigm, rodents are subjected to fixed-time food restriction and have continuous access to running wheels. Under these conditions, animals develop rapid and progressive weight loss, hypophagia, and compulsive wheel running. Contrary to this, animals exposed to either fixed-time food restriction or constant running wheel access maintain body weight indefinitely [13, 18]. Thus, in the ABA paradigm, animals with negative energy balance paradoxically choose to exercise more and eat less, even when food is available. If allowed to continue unchecked, the ABA paradigm results in hypothermia, increased HPA axis activity, and ultimately stomach ulceration and death [13, 19, 20]. Younger animals lose body weight more rapidly than older animals during ABA [21], paralleling clinical observations that AN is a disorder with peak onset and prevalence during adolescence [22, 23].

White adipose tissue (WAT) is an endocrine organ that actively regulates energy balance [24–27]. Weight loss through dieting is frequently followed by weight regain due to significant adaptations in homeostatic systems that control body weight [26–29]. Relapsing into obesity following dieting was recently suggested to be driven by a “metabolic memory” of obesity, which predominantly resides in WAT [24, 26, 30]. For example, gene expression and metabolites within metabolic tissues were compared between lean, obese, or previously obese mice; obesity induced widespread transcriptional and metabolomic changes that were reversible by weight loss across most metabolic tissues, except for WAT [30]. Thus, WAT-driven metabolic memory of obesity has been implicated in weight regain. Furthermore, WAT communicates with the hypothalamus by secreting adipokines and other diffusable factors into the general circulation, while the brain communicates to WAT through sympathetic innervation [31–33]. Finding that interactions between WAT and the hypothalamus regulate body weight during ABA would provide inputs for pursuing obese WAT-derived factors as potential treatment candidates for AN.

Agouti-related peptide (AgRP)-expressing neurons are localized in the arcuate nucleus (ARC) of the hypothalamus and regulate metabolism, food intake, and complex non-feeding behaviors that promote food pursuit [34–38]. For example, AgRP neurons integrate internal signals of energy state with external signals relating to energy state, such as food availability and environmental cues of energy demand, to regulate foraging behavior [39–41]. AgRP neurons also regulate energy expenditure and fuel mobilization based on energy state [42–45]. Recently, neonatal AgRP ablation was reported to increase ABA severity [45], while chemogenetic activation of AgRP neurons reduced ABA progression [45, 46]. Furthermore, activated AgRP neurons shift metabolism towards energy conservation and lipid storage [34, 47–49]. Here, we hypothesized that obese WAT transplants would signal to AgRP neurons to alter the metabolic and/or behavior of recipient mice to protect against weight loss during ABA.

We tested whether transplanting perigonadal WAT from obese HFD-fed mice into normal weight recipient mice would attenuate ABA in the recipients. First, we compared the effects of control versus obese WAT transplant on survival, body weight, food intake, and wheel running in the ABA paradigm. Second, we tested whether neonatal ablation of AgRP-expressing neurons attenuates the ability of obese WAT transplant to reduce weight loss during ABA. We used young adult female mice, since AN predominantly affects young women and girls [1, 50].

## Methods

### Mice

All mice were housed in a climate-controlled room with food and water available *ad libitum* unless otherwise stated. For Experiment 1, mice were maintained on a 12:12 light–dark cycle (lights on at 0700). Weeks before Experiment 2 was conducted, the mouse colony room was switched to a reversed 12:12 light–dark cycle (lights off at 0700). All procedures were approved by the Institutional Animal Care and Use Committee at University of California, San Diego.

All donor and recipient mice were adult females. For Experiment 1, donor and recipient mice were wild type (WT) C57BL/6 J mice purchased from Jackson (JAX) Laboratories. For Experiment 2, donor mice were WT C57BL/6 J from JAX and recipient mice were AgRP-iCre^iDTR+/-^ knockins bred in house. Heterozygous mice expressing Cre recombinase under the control of the AgRP promotor: AgRP-iCre^+/-^ mice, strain AgRP^tm1(Cre)Low1/J^ (012899, JAX) were bred with mice which were heterozygous for a floxed and inactivated simian diphtheria toxin receptor: iDTR mice, strain C57BL/6-Gt(ROSA)26Sor^tm1(HBEGF)Awai/J^ (007900, JAX). Only experimental offspring carrying both the iDTR and AgRP-iCre alleles were susceptible to AgRP neuronal ablation following diphtheria toxin administration.

### AgRP neuron ablation

In Experiment 2, all experimental offspring were injected subcutaneously with diphtheria toxin (50μg/kg) from between P5-P7, as previously described [45, 51]. Diphtheria toxin was dissolved in phosphate-buffered saline (PBS; 13.3 µl/g body weight).

### Immunofluorescence

Following euthanasia, mice were transcardially perfused with PBS containing 4% paraformaldehyde. Brains were removed and post-fixed overnight in 4% paraformaldehyde.

Coronal brain sections (30 μm) were obtained using a vibratome (VT1000P; Leica Microsystems), and sections containing the hypothalamic arcuate nucleus were processed for immunostaining. After being washed for 10 min three times in PBS, the sections were incubated in blocking solution (1:20 normal horse serum in PBS), containing 0.2% Triton X-100 for 1 h at room temperature. Sections were incubated with rabbit anti-AgRP (Phoenix Pharmaceuticals, Lot #: 01826-6) at a dilution of 1:1,000 overnight at room temperature. The next day, sections were washed three times (10 min) in PBS and incubated with goat anti-rabbit Alexa Fluor 488 (Invitrogen, Lot #: ZA386290) at a dilution of 1:1000, 1.5 h at room temperature. The sections were cover-slipped and imaged using an Olympus BX51 fluorescence microscope.

### Adipose tissue transplant

**Fig. 1:**
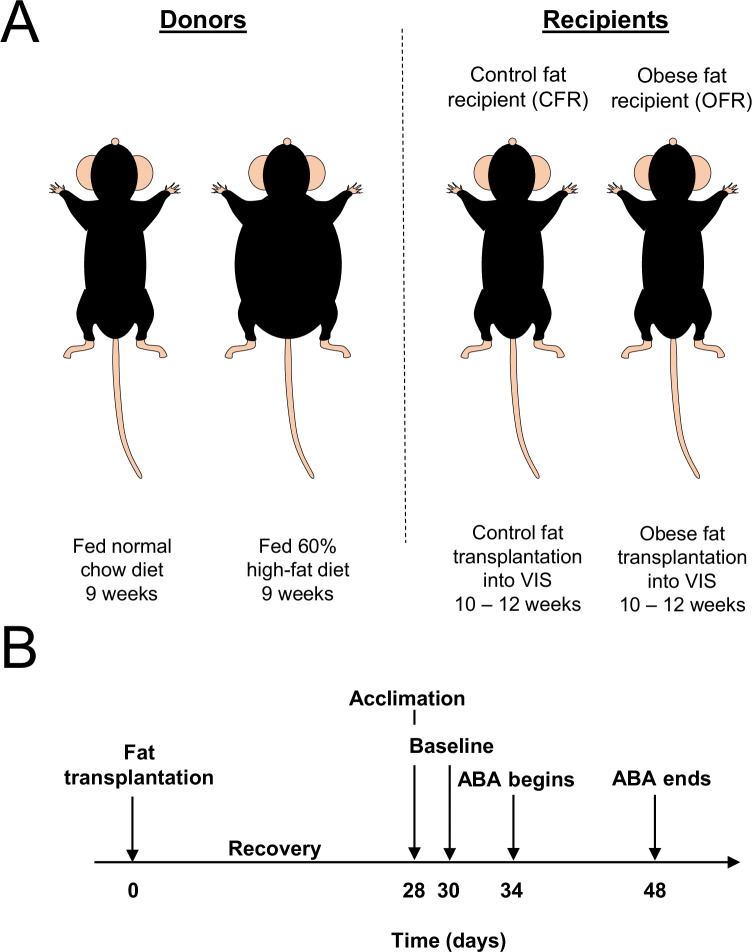
Experimental design. **A** Recipient mice received WAT from either high fat diet (HFD)-fed obese mice or normal chow-fed donor mice. Control or obese WAT was transplanted into the visceral area of normal weight recipient mice. **B** Acclimation to ABA housing conditions began 4 weeks after WAT transplantation. After 2 days of acclimation, 4 days of baseline testing occurred, followed by 2 weeks of testing during restriction conditions.

All experimental mice were randomly assigned to receive WAT transplants from either normal weight or obese donors (Fig. 1A). WAT transplantations were performed as described in Tran et al. [52]. Briefly, beginning at 10 weeks of age, donor mice were fed either HFD (60% calories from fat, Research Diets) to develop obesity, or standardDonors were sacrificed at 19 weeks of age, and intra-abdominal perigonadal WAT was cut into 200 mg slices for obese WAT, or 100 mg for normal weight WAT, and kept in saline-filled 50 ml tubes in a 37 ^o^C water bath until transplantation within 30 min. Double the weight of obese WAT was transplanted compared to normal weight WAT to ensure that equal number of cells were transplanted from both groups, since adipocytes in the obese state are twice as large as normal adipocytes [53]. Perigonadal fat was used for transplants since we reported that the analogous visceral fat pads from male mice exhibit metabolic memory of prior obesity [30]. At 10–12 weeks of age, obese fat recipients (OFR) received obese WAT transplants, while control fat recipient mice (CFR) received WAT from normal weight donors. Recipient mice were anesthetized, and donor WAT slices were sutured next to the mesenteric fat below the liver to increase vascular support. This transplantation method results in vascularized WAT transplants with normal histology compared to endogenous WAT, and without increases of mRNA levels of the inflammatory macrophage surface marker F4/80 or the cytokines IL-6 and TNF-α [52]. After a 4-week recovery, recipient mice were tested in the ABA paradigm (Fig. 1B).

### Activity-based anorexia paradigm

For both experiments, recipient mice 14–16 weeks of age were singly housed in home cages (19.56 × 34.70 × 14.41 cm) in a vivarium room maintained at 70 ± 2 °F. Each cage was equipped with a wireless low-profile running wheel (Med Associates, St Albans, VT, USA), which continuously transmitted running data every 30 s, 24 h a day to a computer with Wheel Manager software. Equipment failure resulted in loss of running wheel data on restriction day 9 of Experiment 2. Food (standard chow; Diet #8604, Inotiv-Teklad) was provided *ad libitum* in a glass jar (5 cm diameter × 4 cm height) resting on the cage floor, and tap water was available *ad libitum* in standard bottles.

Mice were acclimated for 2 days to single housing in the experimental cage. Then, mice entered the baseline phase (4 days), during which standard chow was constantly available, and daily body weight, food intake, and wheel running data were collected. Next, all mice entered the restriction phase, during which food was available for only for 3 h a day beginning at 0900 h. During restriction, mice “drop out” of the paradigm once they lose 25% of their initial body weight which was measured on day 4 of the baseline phase [15, 18, 54]. Mice were sacrificed immediately following drop out [15], or when the study ended at day 14 (Fig. 1B) [15, 18, 54]. Body weight, food intake, and wheel running were recorded daily during both baseline and restriction conditions. The experimenter was blinded to the groups.

No statistical methods were used to determine sample size, but sample sizes were equivalent to previously published studies [29, 45, 55]. For Experiment 1, CFR (n = 21) and OFR (n = 21) mice were assessed in the ABA paradigm. In Experiment 2, CFR and OFR mice additionally underwent either control or neonatal AgRP ablation; thus, four groups of animals were assessed for ABA: CFR intact (n = 12), CFR ablated (n = 14), OFR intact (n = 12), and OFR ablated (n = 13).

### Data analysis

During the restriction phase of ABA, the number of days required for mice to meet the criterion for drop out (loss of 25% of baseline day 4 body weight), termed “survival”, was analyzed using Cox proportional-hazards model (survival analysis). Analysis of the behavioral measures collected during ABA creates statistical challenges due to the presence of expected missing values in the restricted phase, as animals drop out. Thus, general linear mixed models (proc glimmix; SAS v9.2) were used to assess baseline and restriction outcomes, including factors for day, transplant group (control versus obese), and AgRP ablation group (intact versus ablated) for Experiment 2. Separate analyses were performed for body weight, food intake, and wheel running. Food anticipatory activity (FAA, wheel running during the 4-hour period before food delivery), and postprandial activity (PPA, wheel running during the period after food removal until lights off at 1900h) were analyzed in the same fashion. All data analyzed by linear mixed models met the assumptions of the model and equal variance between groups was confirmed prior to analysis. Additionally, prior to analysis, outliers were identified visually, with confirmation by Grubbs’ test. In the case of outliers (1 CFR and 1 OFR in Experiment 1; 1 OFR ablated in Experiment 2), the subject’s data was removed from the entire study. When significant main effects or interactions were observed, post-hoc tests were applied and adjusted for multiple comparisons using the False Discovery Rate (FDR). All tests were two-sided. Alpha levels were set at p < 0.05.

## Results

### Obese WAT transplant reduces weight-loss in the ABA paradigm

**Fig. 2:**
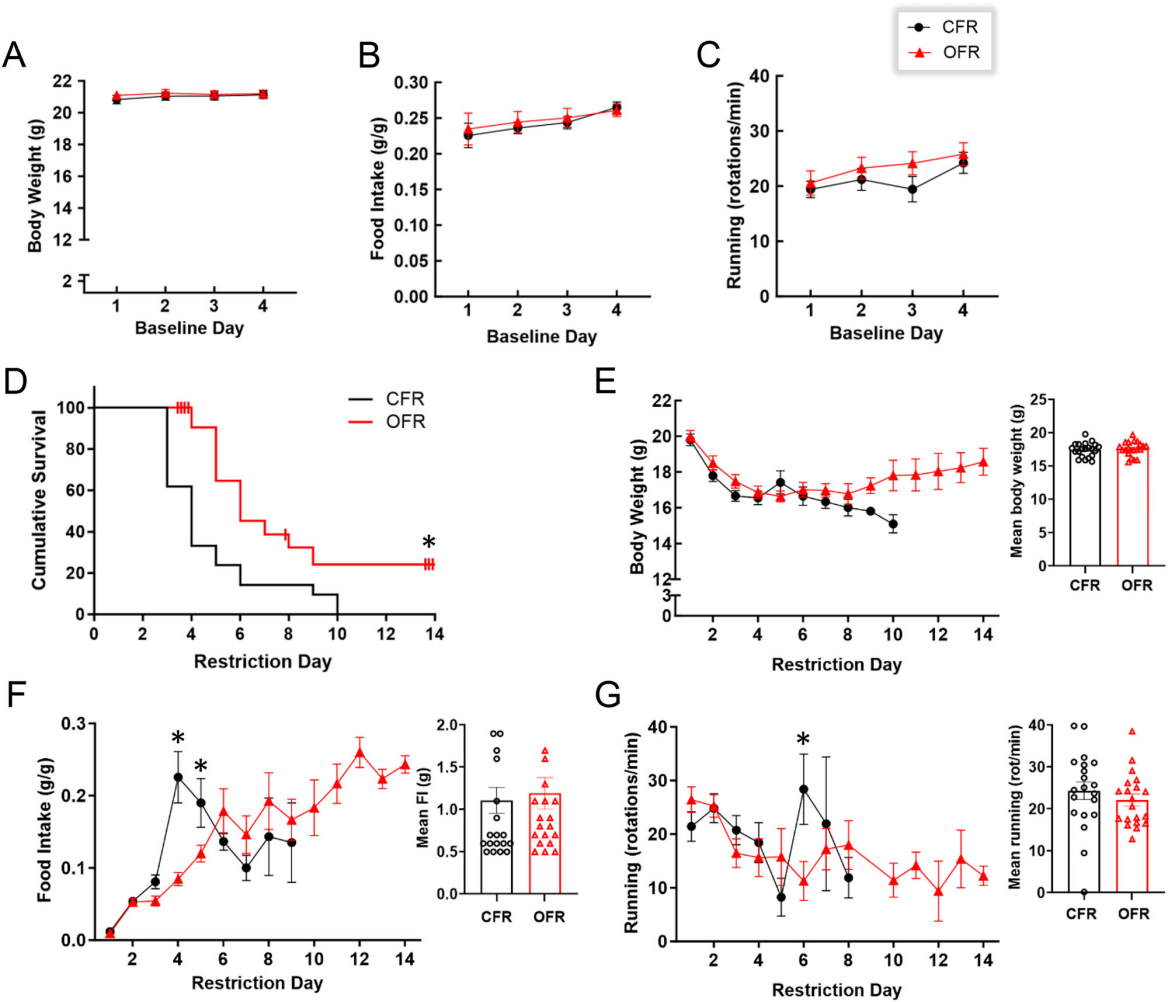
Experiment 1: Effects of obese WAT transplant on ABA. **A** Body weight in grams of CFR and OFR mice over the 4 days. **B** Food intake in grams divided by body weight of CFR and OFR mice over the 4 days. **C** Wheel running revolutions of CFR and OFR mice over the 4 days. **D** OFR mice survived significantly longer than CFR mice during restriction. **E** Body weight of CFR and OFR mice over the 14 days of restriction. **F** CFR mice ate significantly more than OFR mice on days 4 and 5 of restriction. **G** Wheel running of CFR and OFR mice over the 14 days of restriction. Insets show mean values averaged by restriction day. Data are adjusted mean values ± SEM, n = 21/group. An asterisk (*) indicates a significant effect of transplant group.

During the baseline period of Experiment 1, no difference in body weight was found between CFR and OFR mice; however, a main effect of day was observed (F_(3,120)_ = 4.72, p < 0.01; Fig. 2A), with a small increase in body weight emerging over days. Similarly, there was no difference in food intake or wheel running between CFR and OFR mice during baseline (Fig. 2B, C). Additionally, main effects of day were found for food intake (F_(3,120)_ = 2.85, p < 0.05; Fig. 2B) and wheel running (F_(3,114)_ = 5.29, p < 0.01; Fig. 2C) reflecting small increases in these measures over days. Overall, no differences in body weight, food intake, or wheel running were found between CFR versus OFR mice during baseline.

Interestingly, during the restriction period of Experiment 1, OFR mice survived longer in the paradigm than CFR mice (Χ^2^ = 7.51, p < 0.01; Fig. 2D). Hazard ratios indicated that CFR mice were 2.72 times more likely to drop out of the study than OFR mice (95% CI: 1.338–5.596). The longer survival of OFR compared to CFR mice reflects that OFR mice required more days of restriction to reach the 25% weight loss criterion; thus, survival provides one measure of body weight. In sum, obese WAT transplant increased survival under ABA conditions.

Although survival differed between the two transplant groups (Fig. 2D), body weight values for animals still in the study did not differ significantly between CFR and OFR mice during restriction (Fig. 2E). For food intake, a two-way interaction of transplant group and day (F_(8, 137)_ = 8.84, p < 0.001) and post-hoc tests revealed that CFR mice showed a transient increase in consumption relative to OFR mice on day 4 (p < 0.001) and day 5 (p < 0.001) of restriction (Fig. 2F). This sharp increase in food intake by CFR mice coincided with a large number of CFR mice dropping out of the paradigm on days 3 and 4 (Fig. 2D). This transient increase in food intake might reflect the higher food intake values of CFR mice that did not drop out. Regardless of the transient increase in food intake exhibited by CFR mice, this group dropped out of the paradigm significantly faster than OFR mice (Fig. 2D).

For wheel running, there was a two-way interaction of transplant group and day (F_(7, 128)_ = 2.29, p < 0.05). Post-hoc tests revealed that CFR mice showed more running relative to OFR mice only on day 6 of restriction (p < 0.01)(Fig. 2G). It should be noted that in survival studies, the number of mice in the study decreases over time, which can result in a large standard error of the mean as days progress, such as for the CFR group with only 3 mice remaining on day 7 (Fig. 2G). To determine whether transplant groups differed in wheel running during specific phases of ABA, we analyzed wheel running during the FAA and PPA periods. Although main effects of day were observed for both FAA (F_(11,127)_ = 2.88, p < 0.01; Supplemental Fig. 1a) and PPA (F_(13,131)_ = 3.81, p < 0.001; Supplemental Fig. 1b), no main effects or interactions were found for wheel running during the FAA or PPA period.

### AgRP ablation abolishes obese WAT transplant-mediated protection against ABA

**Fig. 3:**
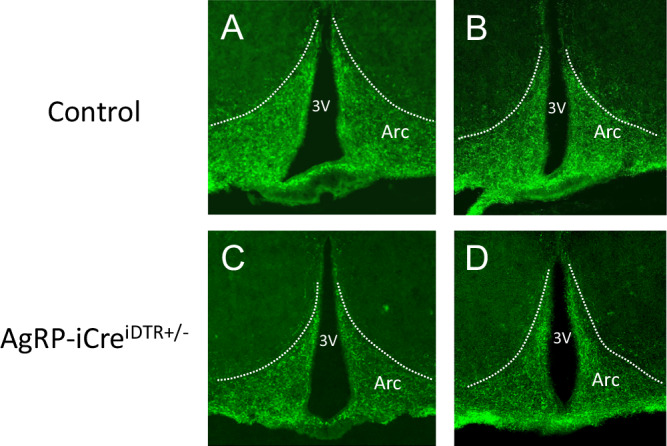
Ablation of AgRP neurons by neonatal diphtheria toxin injections. Female control and AgRP-iCre^DTR+/-^ mice received injections as pups with diphtheria toxin (50ug/kg). Representative immunostaining of ARC neurons of control mice (n = 3) at a more rostral (**A**) and caudal (**B**) level, and AgRP-iCre^DTR+/-^ (n = 3) mice at a more rostral (**C**) and caudal (**D**) level, at 10 weeks of age.

**Fig. 4:**
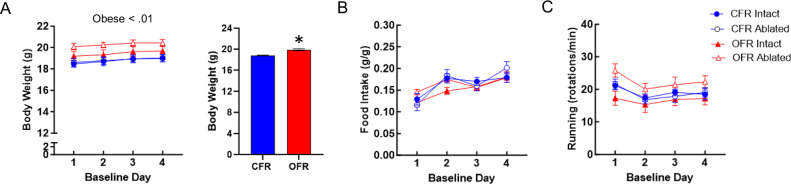
Experiment 2: Effects of obese WAT transplant and neonatal AgRP ablation during baseline. **A** OFR mice weighed more than CFR mice over the 4 days. **B** Food intake in grams divided by body weight of intact or ablated CFR and OFR mice over the 4 days. **C** Wheel running revolutions of intact or ablated CFR and OFR mice over the 4 days. Data are adjusted mean values ± SEM, n = 12–14/group. An asterisk (*) indicates a significant effect of transplant group.

Diptheria toxin injections resulted in mice with ablated AgRP neurons (AgRP-iCre^iDTR+/-^) or intact AgRP neurons (WT^iDTR+/-^)(Fig. 3). In Experiment 2, OFR mice showed a small but significant increase in body weight compared to CFR mice across days during baseline (F_(1, 47)_ = 8.90, p < 0.01; Fig. 4A, B). Further, a main effect of day was observed, with body weight increasing over baseline days (F_(3, 141)_ = 26.70, p < 0.001; Fig. 4A). No difference in food intake or wheel running was found between transplant groups (Fig. 4B, C). Main effects of day were found for both food intake (F_(3,141)_ = 22.87, p < 0.001; Fig. 4B) and wheel running (F_(3,129)_ = 9.60, p < 0.001; Fig. 4C), reflecting small differences over days. Overall, CFR and OFR groups differed only in body weight during baseline, with OFR mice showing slightly higher body weights than CFR mice.

**Fig. 5:**
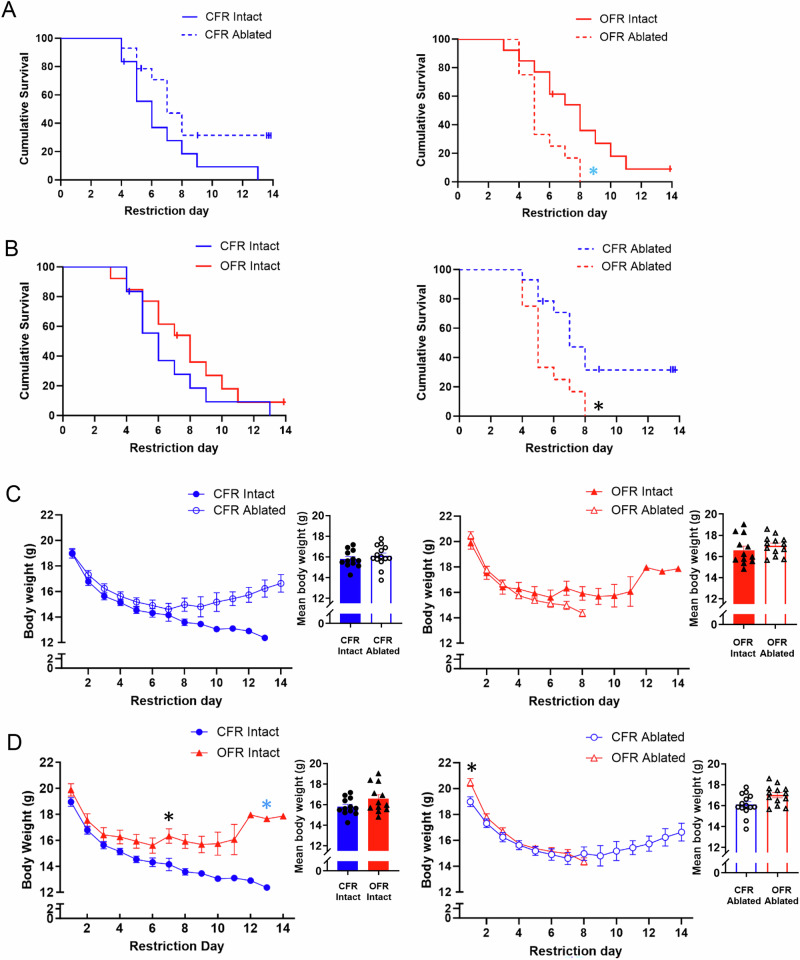
Experiment 2: Effects of obese WAT transplant and neonatal AgRP ablation on survival and body weight during restriction. **A** Ablation reduced survival in OFR, but not CFR mice. **B** Following ablation, OFR mice showed reduced survival compared to CFR mice. **C** No effects of transplant or ablation on body weight. **D** OFR mice showed higher body weight than CFR mice; ablation prevented this effect. Insets show mean values averaged by restriction day. Data are adjusted mean values ± SEM, n = 12–14/group. A black asterisk (*) indicates a significant effect of transplant group. A blue asterisk (*) indicates a trend.

During restriction in Experiment 2, OFR ablated mice showed reduced survival compared to both OFR intact mice (Fig. 5A), and CFR ablated mice (Fig. 5B), as revealed by a transplant and ablation interaction (Χ^2^ = 5.40, p < 0.05)(Fig. 5A, B). Post-hoc hazard ratios indicated that OFR ablated mice dropped out of the paradigm 0.35 times faster than CFR ablated mice (p < 0.01)(Fig. 5B), and 0.49 times faster than OFR intact mice, as indicated by a strong trend (p = 0.05)(Fig. 5B)(95% CI: 1.338–5.596). Within intact mice, transplant group had no effect on survival (p = 0.83)(Fig. 5B). Lastly, ablation group had no effect on survival within CFR mice (p = 0.95)(Fig. 5A).

A three-way interaction of transplant, ablation, and day was found for body weight during restriction (F_(7,253)_ = 2.83, p < 0.01; Fig. 5C, D). Post-hocs revealed that within intact mice, OFR mice had higher body weights than CFR mice, and this effect reached significance on day 7 (p < 0.05 and a trend on day 13 (p = 0.08)(Fig. 5D). Thus, obese WAT transplant increased body weight measures during ABA in both Experiments 1 and 2. Within ablated mice, post-hocs revealed only a small increase in body weight in OFR ablated mice relative to CFR ablated mice on day 1 (p < 0.05)(Fig. 5D). This difference might have resulted from the nonsignificantly higher body weights of OFR ablated mice during baseline (Fig. 4A). Although AgRP ablation reduced survival in OFR mice (Fig. 5A), ablation did not reduce body weight in CFR mice (Fig. 5C). Importantly, in ABA experiments, animals drop out of the paradigm when the weight loss criterion is reached; therefore, some graphed mean values reflect only one animal, such as CFR intact animals (Fig. 5C, D) on days 9–13. In sum, obese WAT transplant increased body weight in intact recipient mice during ABA, but neonatal AgRP ablation prevents this effect.

### Effects of transplant and AgRP ablation on food intake and wheel running during ABA

**Fig. 6:**
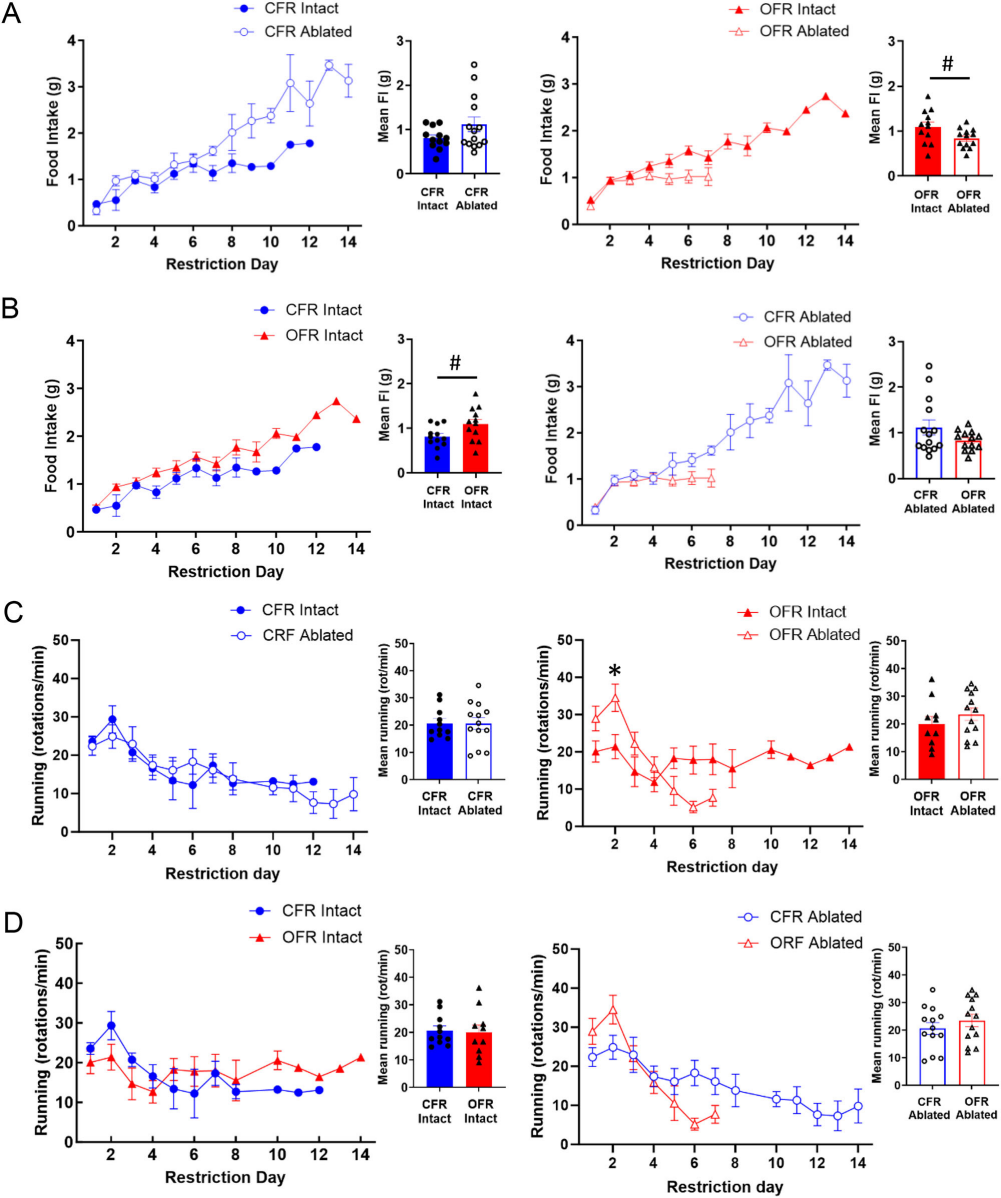
Experiment 2: Effects of obese WAT transplant and neonatal AgRP ablation on food intake and wheel running during restriction. (**A**) A trend for ablation to reduce food intake was found in OFR, but not CFR mice. **B** Following ablation, OFR ablated mice showed a trend for reduced food intake compared to CFR ablated mice. **C** No overall effect of ablation on wheel running of either transplant group. **D** No overall effects of transplant group within control or ablated groups. Insets show mean values averaged by restriction day. Data are adjusted mean values ± SEM, n = 12–14/group. A black asterisk (*) indicates a significant effect of transplant group.

An interaction of transplant and ablation was found for food intake during restriction (F_(1, 47)_ = 5.43, p < 0.05; Fig. 6A, B). However, no interactions including day were found. Post-hoc tests found a trend for OFR intact mice to consume more than both OFR ablated mice (p = 0.09) and CFR intact mice (p = 0.09) across days (Fig. 6A, insets).

For wheel running, an interaction of transplant, ablation, and day was found during restriction (F_(6, 173)_ = 4.09, p < 0.001; Fig. 6C, D). Post-hoc tests showed that OFR ablated mice ran more than OFR intact mice only on day 2. Additional analysis of wheel running during the light versus the dark cycle during restriction revealed no main effects or interactions of transplant, ablation, or day during the light cycle (Supplemental Fig. 3). However during the dark cycle, an interaction of transplant, ablation, and day was found (F_(6, 174)_ = 4.02, p < 0.001; Fig. 6C, D). Post-hoc tests revealed a trend OFR ablated mice to run more than OFR intact mice on days 1 (p = 0.07) and 2 (p = 0.06)(Supplemental Fig. 4). Thus, the increased wheel running of OFR -ablated versus -intact mice early in restriction occurred primarily during the dark cycle, when rodents are most active.

## Discussion

Here we show, for the first time, that transplanting WAT from obese mice into normal recipient mice attenuates the weight loss of recipients in the ABA paradigm. We also show that obese WAT transplant-induced protection against ABA can be prevented by neonatal ablation of AgRP neurons. WAT produces and secretes hormones and exosomes containing other active substances into the bloodstream, thus communicating with other tissues in the body including the hypothalamus [33]. AgRP neurons are known to regulate feeding [34–36], foraging [37, 43], and metabolism [38, 47, 48, 56, 57], and recently, AgRP neurons were reported to sense energy loss and drive feeding, running, and fuel mobilization during the ABA paradigm [45]. Our present findings indicate that AgRP neurons are required for obese WAT transplant to increase body weight maintenance during ABA, an effect that likely involves communication from obese WAT transplants to AgRP neurons through diffusible factors. Our findings that the state of WAT can regulate ABA are consistent with those from the largest GWAS of AN to date, which reported genetic correlations between AN and metabolic, lipid, and anthropometric traits, suggesting that AN is a metabo-psychiatric disorder [8]. Lastly, our results suggest that obese WAT transplant-derived diffusible factors should be examined as potential treatments for AN.

During the 4 day baseline period of the ABA paradigm, we did not observe any consistent effects of WAT transplant or neonatal AgRP ablation. The only exception was a small but significant increase in body weight across baseline days in OFR versus CFR mice. This small effect was observed in Experiment 2 (Fig. 4A), but not Experiment 1 (Fig. 2A). Thus, obese WAT transplant does not reliably increase in body weight under baseline conditions. This small difference in baseline body weight in Experiment 2 did not confound our findings regarding body weight during restriction, since OFR ablated mice had the highest baseline body weight, but showed the most rapid dropout from the ABA paradigm (Fig. 5A, B). Furthermore, our finding that neonatal AgRP ablation does not alter body weight or food intake under baseline conditions is consistent with another report [45, 51].

During the restriction phase of ABA, OFR mice showed increased survival compared to CFR mice in Experiment 1 (Fig. 2D), and increased body weight compared to CFR mice in Experiment 2 (Fig. 5D). Thus, obese WAT transplant increased body weight measures during ABA in both studies. While the survival measure indicates the day by which mice have lost 25% of their baseline day 4 body weight, the body weight measure indicates the average weight of mice still remaining in the paradigm. Thus, these two measures capture different aspects of body weight loss during the restriction period. In sum, the results of the two studies both indicate that obese WAT transplant reliably reduces weight loss in intact mice during the restriction period of ABA.

One report suggests that normal mice receiving AgRP ablation during the neonatal period show substantial reductions in survival, body weight, food intake, and wheel running during the restriction phase of ABA [45]. However, we did not observe any of these effects of ablation in CFR mice during restriction (Figs. 5A, C and 6A, C), including survival (p = 0.95)(Fig. 5A). Our present studies tested 14 week-old mice using a 3 h period of food access during restriction, while previous work tested 5–7 week-old mice using a 2 h period of food access [45]; these methodological differences may account for the discrepancy in results, since longer periods of food availability and the use of older animals both substantially reduce weight loss and increase survival during ABA, and could have obscured effects of ablation [18, 21]. Our results also suggest that obese WAT transplant might increase the role of AgRP signaling in maintaining body weight. For example, obese WAT-derived diffusible factors might exert a stronger effect on AgRP signaling than control WAT. Indeed, control and obese WAT show different patterns of gene expression, resulting in differences in secreted factors [30, 58, 59]. In support of this idea, neonatal AgRP ablation reduced survival in OFR, but not CFR mice (Fig. 5A). Lastly, a small increase in body weight observed in OFR ablated mice on the first day of restriction likely resulted from the small increase in body weight observed in this group during baseline (Fig. 4A). In sum, our findings suggest that AgRP neurons are essential for the ability of obese WAT transplant to prevent weight loss during ABA.

In intact mice, obese WAT transplant showed a trend to increase food intake compared to CFR mice, as well as OFR ablated mice, in Experiment 2 (Fig. 6A, B). This increase in food intake may underlie the longer survival time of OFR intact mice relative to OFR ablated mice (Fig. 6A), and the higher body weight of OFR intact mice relative to CFR intact mice (Fig. 6D). However, OFR did not show increased food intake in Experiment 1. The dramatically faster rate of drop out of CFR mice in Experiment 1 might have obscured detection of reduced food intake in this group. For example, a sharp but transient increase in food intake was observed in CFR mice relative to OFR mice on days 4 and 5 of restriction (Fig. 2F) in Experiment 1. However, food intake rapidly became comparable between the transplant groups on the remaining days. This transient increase in food intake in CFR mice likely emerged due to the rapid drop out of over 60% of CFR mice on days 3 and 4, and the large food intake values of the few remaining CFR mice. The observed trend for OFR ablated mice to eat less during restriction is consistent with our previous work showing that obese WAT transplants contain specific factors that can encourage feeding [30].

Neither obese WAT transplant nor neonatal AgRP ablation altered wheel running during restriction overall, or during the FAA or PPA periods (Supplemental Figs. 1 and 2). In Experiment 1, CFR mice showed increased running only on day 6, when few animals remained in the paradigm (Fig. 2G). In Experiment 2, only a small increase in running was observed in OFR ablated versus OFR intact mice on day 2 of restriction, which might have resulted from the nonsignificant increases in running by this group during baseline (Fig. 4C). Robust FAA was not observed in the present studies. This finding could be due to the use of C57Bl6/J female mice, which show slower onset of FAA during restriction than C57Bl6/J males [60], or could be due to the change in light cycle during the FAA period. Overall, our present findings suggest that increases in food intake, rather than changes in running, underlie the longer survival times and greater body weights of OFR intact mice during ABA.

Leptin is a hormone secreted by WAT that is central to adipose-hypothalamic signaling. In our present studies, obese WAT transplants may have secreted more leptin than control WAT transplants, for several reasons. First, HFD increases leptin secretion from perigonadal fat [61], and mice with HFD-induced obesity show approximately 10-fold higher plasma leptin levels compared to lean controls [62]. Chronic leptin administration to rats during ABA reduces wheel running, but has no effect in rats exposed only to running wheels, only food restriction, or neither condition [63, 64, 65]. Furthermore, chronic leptin administration reduces food intake in these four conditions, and does not increase bodyweight in any of these conditions[63]. Given our present findings that obese WAT transplant prevented weight loss during ABA, (Figs. 2D and 5D), this effect is likely leptin-independent.

Adipose tissue communicates with the brain through factors secreted into the bloodstream [31, 32]. Secreted factors derived from transplanted obese WAT and/or associated stromal vascular cells likely interact with AgRP neurons to reduce ABA progression [30]. Indeed, activated AgRP neurons shift metabolism towards energy conservation and lipid storage through several mechanisms including reduction of energy expenditure, elevated carbohydrate utilization, and reduced sympathetic outflow [34, 48, 49, 66]. We previously identified “metabolic memory” genes, which are obesity-induced genes that are persistently dysregulated even after weight loss. Using a diet switch model, we compared gene expression of epididymal WAT from mice fed high-fat diet (HFD) switched to low fat diet (LFD), to mice maintained only on HFD or LFD. Using rna-seq, we found that obesity-induced genes which were persistently dysregulated even after weight loss were localized primarily in adipocytes [24, 30]. Genes we identified included ATP6v0a1, a subunit of the vacuolar H + ATPase, a large multi-subunit enzyme proton pump that is involved in the regulation of intracellular pH homeostasis [67], prostaglandin D2, and hematopoietic prostaglandin D synthase [24]. However, these diet switch studies were performed in male mice, while our present studies were performed in female mice. Future studies should identify metabolic memory genes using female mice to identify targets for novel therapeutic development to prevent weight loss in AN [68–71].

Other work has identified molecular signaling mechanisms within AgRP neurons that are altered by diet- or genetically-induced obesity [72–75], which may also provide clues for future studies examining mechanisms underlying the present findings. Identifying factors secreted by obese WAT transplants, and resultant alterations to AgRP neuronal function, that increase body weight maintenance during ABA may inform novel treatment approaches for AN. Lastly, our findings also suggest that autologous WAT transfer following reprogramming to reflect an obese state should be explored as a potential treatment for AN.

The present studies have several caveats. We used female mice as both donors and recipients, since predominantly girls and women develop AN. Future preclinical work should be extended to males, as boys with AN present with cognitive and behavioral symptoms that are as severe as female patients [76]. Further, numerous sex differences have been reported in WAT function in humans, including adipogenesis, the storage and release of fatty acids, and secretory function [77–79]. Another limitation of the present studies is that neonatal ablation of AgRP neurons induces compensatory changes in neural networks which may complicate the interpretation of results. Early disruption of AgRP neurons has been reported to induce compensations within reward circuitry (Dietrich et al. [80]). For example, both neonatal ablation of AgRP neurons and cell-specific deletion of *Sirt1* in AgRP neurons increase responses to cocaine during adulthood (Dietrich et al.) [80]. Finally, AgRP constitutive knock mice do not display obvious feeding or body weight deficits and maintain a normal response to starvation, demonstrating that AgRP is not a required orexigenic factor, and other pathways can regulate energy homeostasis can compensate for the loss of AgRP (Qian et al.) [81]. However, ablation of AgRP neurons in adult animals results in death, precluding the use of this approach [51]. Another limitation is that, like other studies using AgRP neuron ablation [45, 51, 82], we used mice harboring a heterozygous, rather than homozygous, allele for DTR expression, to permit ablation of AgRP neurons upon DTR activation following diphtheria toxin injection (Fig. 3). However, the use of mice carrying a heterozygous rather than a homozygous DTR allele has been shown to result in less complete AgRP ablation [45]. Nevertheless, our present results show that AgRP-iCre^iDTR+/-^ mice receiving obese WAT transplant and neonatal diphtheria toxin injections exhibit some residual AgRP staining (Fig. 3), but also demonstrate robust reductions in survival compared to their nonablated counterparts (Fig. 5A).

Future studies using chemogenetics to inhibit AgRP neurons only during ABA would complement our present work; however, chronic neuromodulation using designer receptors activated by designer drugs (DREADDs) has potential drawbacks, including DREADD desensitization and loss of inhibitory control of targeted neurons [83–85]. Finally, our findings indicate that AgRP neurons are essential for obese WAT transplant to reduce ABA progression; however, it is possible that additional hypothalamic neuron subtypes, including alpha-melanocyte stimulating hormone containing neurons, are also required for this effect.

In summary, transplanting WAT from an obese donor into a normal weight recipient attenuates weight loss during ABA in the recipient, and neonatal AgRP ablation prevents this effect. The increased body weight exhibited by OFR mice during ABA is associated with a trend to increase feeding, and may also involve metabolic changes. Our results strongly suggest that obese WAT transplant-derived factors communicate with AgRP neurons, resulting in increased body weight maintenance during ABA. These findings provide essential groundwork for the identification of obese WAT-derived factors for the development of pharmacological treatments for AN, the deadliest psychiatric disorder.

## Supplementary information

Supplementary Figure 1.

Supplementary Figure 2.

Supplementary Figure 3.

Supplementary Figure 4.

## Data Availability

The data that were used to support the findings of this study are available from the corresponding author upon request.
