# Supplementary material for: The mitigation of activity-based anorexia by obese adipose tissue transplant is abolished by neonatal AgRP neuron ablation
Source: Transl Psychiatry. 2026 Mar 23;16:199. doi: 10.1038/s41398-026-03970-2 (PMC13040072; doi:10.1038/s41398-026-03970-2)
Supplement: Supplementary file 1 — Supplementary Figure 1. [file 41398_2026_3970_MOESM1_ESM.pdf]

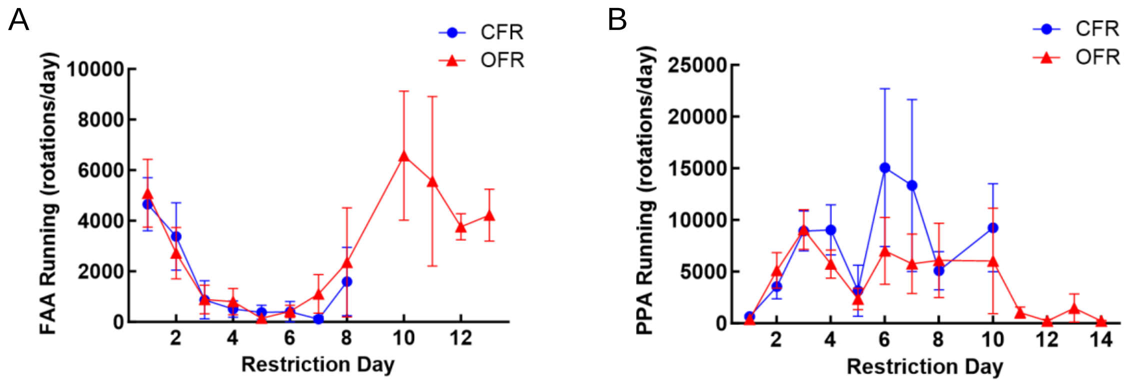

Supplementary Figure 1. Experiment 1: Transplant had no effect on wheel running during the FAA (A) or PPA (B) period of restriction. Data are adjusted mean values ± SEM, n = 21/group.
