# Supplementary material for: The mitigation of activity-based anorexia by obese adipose tissue transplant is abolished by neonatal AgRP neuron ablation
Source: Transl Psychiatry. 2026 Mar 23;16:199. doi: 10.1038/s41398-026-03970-2 (PMC13040072; doi:10.1038/s41398-026-03970-2)
Supplement: Supplementary file 3 — Supplementary Figure 3. [file 41398_2026_3970_MOESM3_ESM.pdf]

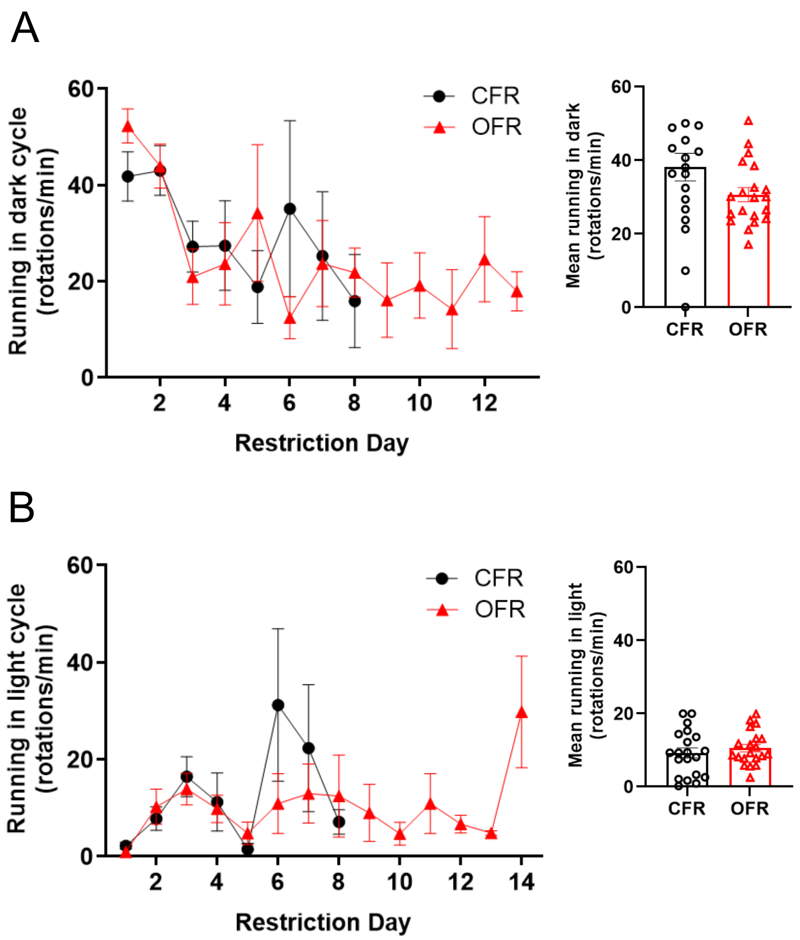


**Supplementary Figure 3.** Experiment 1: Transplant had no effect on wheel running during the dark cycle (A) or the light cycle (B) of the restriction period. Insets show mean values averaged by restriction day. Data are adjusted mean values ± SEM, n = 21/group.
