# Supplementary material for: The mitigation of activity-based anorexia by obese adipose tissue transplant is abolished by neonatal AgRP neuron ablation
Source: Transl Psychiatry. 2026 Mar 23;16:199. doi: 10.1038/s41398-026-03970-2 (PMC13040072; doi:10.1038/s41398-026-03970-2)
Supplement: Supplementary file 4 — Supplementary Figure 4. [file 41398_2026_3970_MOESM4_ESM.pdf]

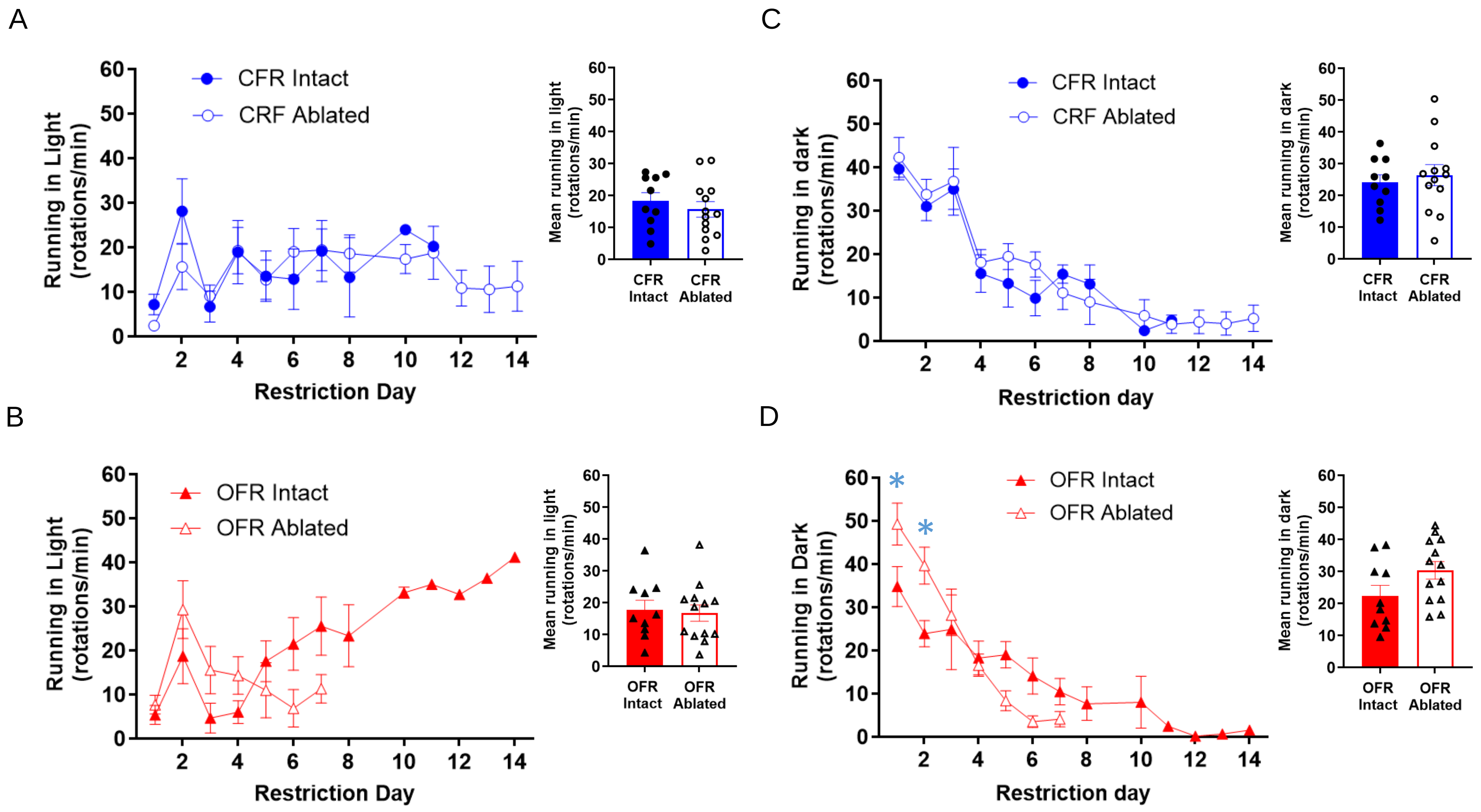

Supplementary Figure 4. Experiment 2: Neither transplant nor ablation altered wheel running during the light cycle (A,B) or the dark cycle (C,D) during the restriction period. During the dark cycle, a trend was found for AgRP neuron ablation to increase running during the dark cycle on days 1 and 2. Insets show mean values averaged by restriction day. Data are adjusted mean values ± SEM, n = 12-14/group. A blue asterisk (*) indicates a trend.
